# Clinical diagnosis and treatment of a patient with hypertension, hypokalemia, and bilateral adrenal adenomas

**DOI:** 10.3389/fendo.2025.1558841

**Published:** 2025-05-09

**Authors:** Tingting Zhang, Tao Chen, Wenjie Zhang, Lu Tan, Li Li, Yuchun Zhu, Yan Ren

**Affiliations:** ^1^ Department of Health Management Center, General Practice Medical Center, West China Hospital, Sichuan University, Chengdu, Sichuan, China; ^2^ Department of Endocrinology and Metabolism, Adrenal Center, West China Hospital, Sichuan University, Chengdu, Sichuan, China; ^3^ Department of Nuclear Medicine, West China Hospital, Sichuan University, Chengdu, Sichuan, China; ^4^ Institute of Clinical Pathology, West China Hospital, Sichuan University, Chengdu, Sichuan, China; ^5^ Department of Urology, West China Hospital, Sichuan University, Chengdu, Sichuan, China

**Keywords:** bilateral aldosterone-producing adenomas, subtyping diagnosis, 68 Ga-Pentixafor PET/MR, CYP11B2, bilateral partial adrenalectomy

## Abstract

Aldosterone-producing adenoma (APA) is one of the main types of primary aldosteronism (PA). APAs are predominantly unilateral, whereas bilateral APAs are rare. Currently, clinical experience in the diagnosis and treatment of bilateral APAs is limited, posing significant challenges. This article reports the case of a PA patient with bilateral adrenal nodules. Adrenal venous sampling (AVS) revealed no dominant lateral secretion, but ^68^Ga-Pentixafor PET/MR imaging, which targets CXCR4, revealed bilateral positive lesions. The patient achieved biochemical and clinical remission after undergoing bilateral partial adrenalectomy, and CYP11B2 immunohistochemical staining confirmed that both nodules were APAs. This case report suggests that ^68^Ga-Pentixafor nuclear imaging, which targets CXCR4, is a noninvasive and reliable method for PA subtyping and may be the optimal method for the definitive diagnosis of bilateral APA. Bilateral partial adrenalectomy may be an effective and safe surgical procedure for the treatment of bilateral APAs. This study provides new insights and considerations for clinicians in the diagnosis and treatment of bilateral APAs.

## 1 Introduction

Primary aldosteronism (PA) is the most common cause of secondary hypertension (1). Aldosterone-producing adenoma (APA) and idiopathic hyperaldosteronism (IHA) are the main types of PA, accounting for 35% and 60% of PA cases, respectively. Accurate subtyping is extremely challenging and crucial for the selection of treatment. APAs are predominantly unilateral, whereas bilateral APAs are rare, and there is currently limited clinical experience in their diagnosis and treatment. CT fails to elucidate the secretory function of the detected nodules in bilateral APAs, and adrenal venous sampling (AVS) can only determine whether there is unilateral dominant secretion in bilateral lesions but cannot accurately differentiate bilateral APAs from IHA with nonfunctional adenomas (NFAs) (2, 3). Research has revealed that CXC chemokine receptor type 4 (CXCR4) is highly expressed on APA cell membranes and is strongly correlated with CYP11B2 expression levels (4). ^68^Ga-labeled Pentixafor, a CXCR4-specific ligand, enables functional imaging with PET/CT, providing guidance for subtyping diagnosis and clinical decision-making in PA (4–7). In this paper, we report a case of a PA patient with bilateral adrenal nodules. AVS revealed no dominant lateral secretion, but targeting CXCR4 with ^68^Ga-Pentixafor PET/MR imaging revealed bilateral positive lesions. The patient underwent bilateral partial adrenalectomy followed by final pathology, which confirmed that both nodules were APAs.

## 2 Case presentation

The patient, a 44-year-old female, was admitted to the hospital because of elevated blood pressure and decreased serum potassium for an 11-year duration.

Eleven years previously, the patient presented with dizziness and headache, and upon examination, elevated blood pressure was detected. CT revealed a right adrenal nodule, and the patient underwent resection of the nodule in the right adrenal gland at the local hospital (the patient was unable to provide relevant medical records). Postoperatively, the patient’s blood pressure fluctuated from 140–160/90–100 mmHg, peaking at 200/130 mmHg, accompanied by hypokalemia, with the lowest level reaching 1.9 mmol/L. Treatment with 30 mg nifedipine once a day, 150 mg irbesartan once a day, and 40 mg spironolactone three times a day failed to adequately control blood pressure and serum potassium levels.

Seven years prior, the patient presented to our hospital. Following the discontinuation of irbesartan and spironolactone for one month and correction of hypokalemia, the patient’s blood biochemistry tests revealed a significant increase in the aldosterone-to-renin ratio (ARR); plasma aldosterone was not suppressed in the saline infusion test or the captopril challenge test (**Table 1**). No significant abnormalities were detected in the adrenocorticotropic hormone (ACTH) levels, cortisol circadian rhythm, 24-hour urine-free cortisol levels, 1-mg overnight dexamethasone suppression test, low-dose dexamethasone suppression test, blood and urine catecholamines and metabolites, sex hormones, or 17α-hydroxyprogesterone. Consequently, the patient was definitively diagnosed with PA. Adrenal CT revealed low-density nodule shadows in both adrenal glands, measuring 19×10 mm on the right side and 17×12 mm on the left side. AVS without ACTH stimulation revealed no dominant lateral secretion (**Table 2**). The patient was diagnosed with IHA and treated with spironolactone and amlodipine benzenesulfonate tablets. During follow-up at a local hospital, the patient was reviewed at our hospital five and three years prior, both of which revealed significantly elevated ARR, poor blood pressure control and persistent hypokalemia, without significant enlargement of bilateral adrenal nodules. One and a half years ago, the patient developed breast hyperplasia and a left breast mass. A needle biopsy of the mass in the left breast revealed a benign lesion.

**Table 1:** Results of screening and confirmatory testing.

Screening/Confirmatory test	DRC (µIU/mL)	PAC (ng/dL)	ARR
Screening	4.59	70.75	15.41
Pre SIT	<0.82	38.83	/
Post SIT	<0.82	37.88	/
Pre CCT	21.32	49.17	2.31
Post CCT	23.94	45.04	1.88

SIT, Saline infusion test; CCT, Captopril challenge test; DRC, direct renin concentration; PAC, plasma aldosterone concentration; ARR, aldosterone-to-renin ratio.

**Table 2:** Results of adrenal venous sampling without ACTH stimulation.

Sampling sites	Cor (nmol/L)	PAC (ng/dL)	PAC/Cor	LI
Inferior vena cava	157.1	41.14	0.26	/
Left adrenal vein	1204	280.8	0.23	1.05
Right adrenal vein	1311	286.6	0.22	/

Cor, cortisol; PAC, plasma aldosterone concentration; LI, lateralization index (the ratio of PAC/Cor in the dominant side divided by that in the nondominant side).

**Figure 1:**
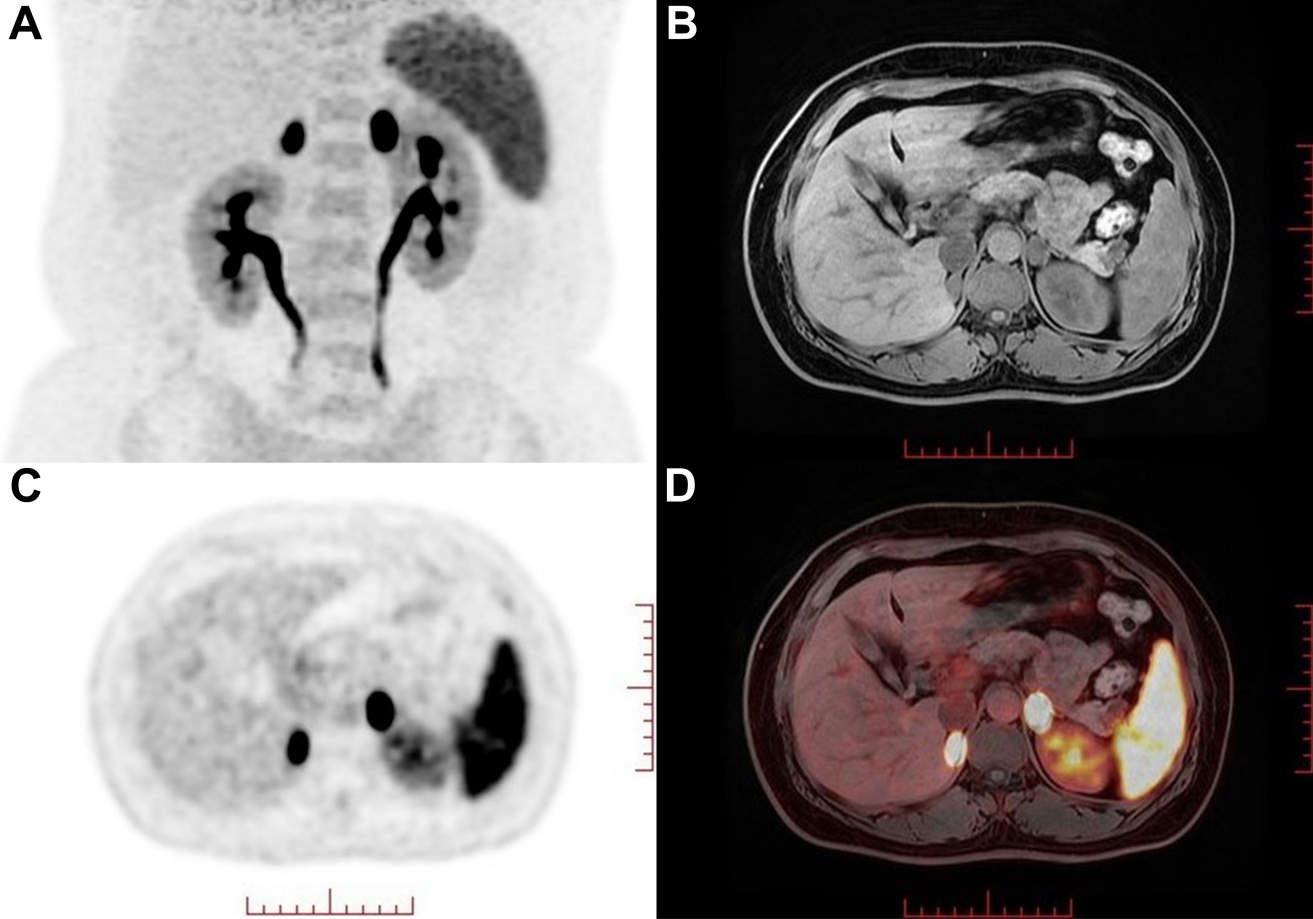
^68^Ga-Pentixafor PET/MR nuclear imaging. **(A)** MIP map showed foci in bilateral adrenal areas. **(B-D)** MRI and fusion images showed a 19×15 mm (right) and 18×16 mm(left) nodule with intense uptake (SUVmax values of 29.1 and 46.0 on the right and left sides, respectively) in each of the adrenal medial limbs of the bilateral adrenal glands.

**Figure 2:**
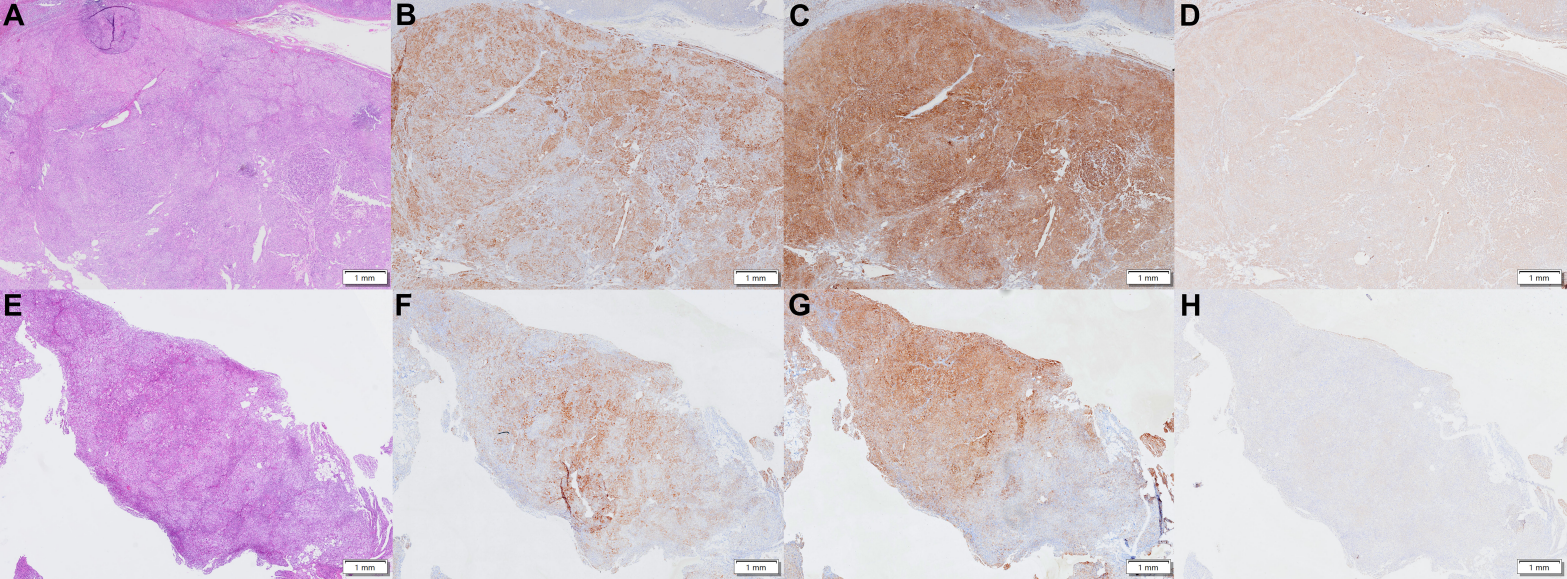
Histological and immunohistochemical examination results. Immunohistochemistry revealed diffuse positive expression of CYP11B2 and CXCR4 in both adrenal tumor tissues, with mild positive expression of CYP11B1 in the left adrenal tumor tissue. **(A-D)** left adrenal tumor tissue. **(E-H)** right adrenal tumor tissue. **(A, E)** H&E staining. **(B, F)** immunohistochemical staining for CYP11B2. **(C, G)** immunohistochemical staining for CXCR4. **(D, H)** immunohistochemical staining for CYP11B1. Magnification ×8 for immunohistochemical staining.

One year earlier, the patient revisited our outpatient clinic with a blood pressure fluctuating from 150–160/90–100 mmHg and a serum potassium level of 2.16–2.94 mmol/L. To specify the functional status of the adenomas on both sides, the patient underwent CXCR4 targeting with ^68^Ga-Pentixafor PET/MR imaging. The results revealed mixed signal nodules in each of the adrenal medial limbs of the bilateral adrenal glands, measuring 19×15 mm on the right side and 18×16 mm on the left side. The nodules on both sides exhibited abnormally increased uptake of ^68^Ga-Pentixafor, with SUVmax values of 29.1 (right side) and 46.0 (left side) (**Figure 1**). The liver SUVmax was 1.8, and the SUVmean was 1.4. The right-sided lesion-to-liver ratio (LLR) was 20.9, and the left-sided LLR was 33.1. These findings suggested that both bilateral adrenal nodules were APAs. The patient subsequently underwent bilateral partial adrenalectomy. On the second postoperative day, the patient’s ACTH level was 16.59 ng/L (reference range: 5.00–78.00), the direct renin concentration (DRC) was <0.5 µIU/mL (reference range: 4.40–46.10), the plasma aldosterone concentration (PAC) was 2.33 ng/dL (reference range: 3.00–35.30), and the serum potassium level was 3.44 mmol/L (reference range: 3.50–5.30). Postoperatively, hydrocortisone was intravenously injected to avoid adrenal insufficiency, which was subsequently replaced by oral prednisone acetate tablets, with a gradual reduction in dosage until discontinuation. Postoperative pathological analysis of the bilateral adenomas confirmed the presence of cortical adenomas. Immunohistochemistry revealed diffuse positive expression of CYP11B2 and CXCR4 in both adrenal tumor tissues (**Figure 2**). The patient was ultimately diagnosed with PA caused by bilateral APA.

Following surgery, the patient discontinued the use of antihypertensive medication, and her blood pressure and serum potassium level remained normal. At seven months postsurgery, the serum potassium level was 4.29 mmol/L, the DRC was 10.37 µIU/mL, the PAC was 4.07 ng/dL, the ARR was 0.39, the ACTH level was 33.82 ng/L, and the cortisol level at 8:00 AM was 255 nmol/L. At the 14-month postoperative telephone follow-up, no antihypertensive medication was taken by the patient. Her blood pressure (120–130/80–90 mmHg), serum potassium level, ARR, and renal function were all within normal ranges. The left breast mass was slightly smaller in size than previously reported. According to the PASO criteria, the patient achieved complete clinical and biochemical remission (8).

## 3 Discussion

To our knowledge, the present case is the first report of AVS combined with ^68^Ga-Pentixafor PET/MR targeting CXCR4 and the conclusive confirmation of bilateral APA by CYP11B2 immunohistochemical staining. Previously reported APAs have been overwhelmingly unilateral, whereas bilateral APAs are rare, and currently, the exact prevalence and clinical features of the latter are still unknown. Wu et al. (9) identified 7 cases of bilateral APA among 164 patients diagnosed with APA by histopathology, with a distribution of 4.3%, suggesting that bilateral APA is not a rare disease.

The clinical diagnosis and treatment of bilateral APA pose challenges. Traditional subtyping methods, such as CT, cannot provide functional information for bilateral lesions. At present, AVS is still the main method recommended by guidelines or consensuses for determining the dominant lateral secretion in cases of PA (10, 11). However, in addition to their intrinsic limitations, such as invasiveness, radiation exposure, high operational technical requirements, lack of standardized procedures, and difficulties in interpreting results, recent studies have reported recurrence or failure to achieve a biochemical cure after unilateral adrenalectomy in patients with a unilateral dominant side demonstrated by AVS (8, 12). Furthermore, theoretically, successful AVS performed in a patient with bilateral APA may occur in two conditions: if the bilateral adenomas are functionally equivalent, the result of AVS will be considered to have no dominant side; if the bilateral adenomas display asymmetric autonomous secretion, the AVS result may be construed as indicating an adenoma on one side and a nonfunctional tumor on the other. Morimoto et al. (13) reported a patient with bilateral adrenal nodules. On the basis of the results of AVS performed via the bilateral adrenal central veins (C-AVS), the patient could have been possibly diagnosed with unilateral PA (lateralization index, LI = 4.71). However, segmental AVS (S-AVS) performed by sampling adrenal effluents from tributaries confirmed the presence of bilateral APAs, revealing asymmetric secretion of bilateral APA function. The patient in this case had a confirmed diagnosis of PA, and CT revealed bilateral adenomas. In accordance with the guidelines, AVS should be performed to clarify the dominant side, and the results suggest that there is no dominant lateral secretion (LI = 1.05). Based on traditional thought, the patient was initially diagnosed with IHA. Therefore, AVS has significant limitations for the precise diagnosis of bilateral APAs and cannot be used as a subtyping diagnostic method for these tumors.

In 2018, Heinze et al. (4) reported that CXCR4 was highly expressed on APA cell membranes and was strongly correlated with the expression of CYP11B2. Since then, several studies have shown that targeting CXCR4 with ^68^Ga-Pentixafor PET/CT imaging has assisted in the diagnostic subtyping of PA. Ding et al. (5) reported that the sensitivity and specificity of visual assessment using ^68^Ga-Pentixafor PET/CT in patients with PA were 97.8% and 87.5%, respectively. The specificity increased to 88.2% when a cutoff value for LLR of ≥ 2.5 was used. Zheng et al. (6) further conducted a larger sample size study, revealing that the sensitivity and specificity of ^68^Ga-Pentixafor PET/CT for visual analysis in diagnosing APA were 92.40% and 94.40%, respectively. For nodules with a diameter of ≥1 cm and a SUVmax of ≥7.3, the specificity was 100%. Hu et al. (7) reported that the concordance rate between ^68^Ga-Pentixafor PET/CT and AVS in lateralizing PA reached 90%, and almost all (86%) had a discrete unilateral adenoma, demonstrating the high accuracy of ^68^Ga-Pentixafor PET/CT in identifying unilateral APAs. In this case, despite previous AVS revealing no dominant lateral secretion, the patient presented with severe hypertension accompanied by hypokalemia. Treatment with high-dose spironolactone was ineffective. CT revealed solitary nodules 1–2 cm in diameter on both adrenal glands, suggesting the possibility of bilateral hyperfunctioning adenomas. Accordingly, we recommended that the patient undergo ^68^Ga-Pentixafor PET/MR imaging. The results revealed that the uptake of ^68^Ga-Pentixafor by the bilateral adrenal nodules was abnormally increased. The SUVmax values of the bilateral positive lesions were 29.1 (right side) and 46.0 (left side). The right-sided LLR was 20.9, and the contralateral LLR was 33.1. On the basis of these findings, the diagnosis of bilateral APA can be unequivocally confirmed through either visual assessment or semiquantitative analysis.

From this case, it can be concluded that for patients with PA presenting typical clinical features of APA (hypertension, hypokalemia, PAC >20 ng/dL), if CT reveals bilateral solitary adrenal nodules 1–2 cm in diameter, the possibility of bilateral APA should be considered. When selecting a subtyping method, ^68^Ga-Pentixafor nuclear imaging, which targets CXCR4, is more helpful than AVS in assisting with the subtyping diagnosis for such patients.

It should be noted that a minority of studies have revealed that NFA may result in false-positive uptake on ^68^Ga-Pentixafor PET/CT (14, 15). For bilateral adrenal nodules, there may be an APA on one side and an NFA with false-positive uptake on the other. Hu et al. (7) reported that no patients with bilateral PA would be misdiagnosed as having unilateral PA when the cutoff value of LI based on the SUVmax at 10 minutes was 1.65, with a specificity of 100% and a sensitivity of 77.00%. Another prospective study revealed that the diagnostic performance of LLR for unilateral PA surpassed that of the SUVmax, LCR, and AVS-LI. At an LLR cutoff value of 3.05, the specificity for identifying functional nodules reached 100%, with a sensitivity of 94.74% (16). Therefore, when using ^68^Ga-Pentixafor PET/CT for the subtyping of bilateral adrenal nodules, it is necessary to incorporate semiquantitative parameters alongside visual assessment to minimize the impact of false-positive uptake on subtyping diagnosis; AVS can be considered when necessary.

At present, there is no consensus on the treatment of bilateral APA, and the choice of treatment options for this patient is worth exploring. Current consensus recommends total adrenalectomy of the lesion side for unilateral APA, whereas pharmacological therapy is preferred for bilateral lesions. Satani et al. (17) and Satoh et al. (18) proposed that S-AVS can help design a surgical approach to completely remove tumors while preserving nontumor adrenal tissues in patients with bilateral APAs. However, its widespread clinical application has been limited by high technical requirements and high costs. The removal of both adrenal glands significantly increases the likelihood of adrenal insufficiency, necessitating lifelong glucocorticoid replacement therapy. Therefore, partial adrenalectomy may serve as an alternative that achieves effective treatment while maximally preserving adrenal cortex function. Williams et al. (19) retrospectively assessed the postsurgical outcomes of 13 patients with bilateral PA who underwent adrenalectomy (partial or total), of whom 5 patients were diagnosed with bilateral APA by histopathological analysis. Their complete clinical and biochemical success rates at 6 to 12 months of postsurgical follow-up were 60% (3/5) and 100% (5/5), respectively. Nanba et al. (20) reported a patient with bilateral APA who underwent bilateral partial adrenalectomy and achieved cure without the need for glucocorticoid replacement therapy. On the basis of the above findings, we established a treatment protocol involving laparoscopic bilateral partial adrenalectomy. Postoperative pathology ultimately confirmed that both adenomas were APAs, and postoperative follow-up revealed that the patient’s blood pressure, serum potassium level, DRC, and PAC had returned to normal. Renal function was also found to be intact. The complete discontinuation of antihypertensive medication and potassium supplementation suggested that the patient achieved complete clinical and biochemical remission. Therefore, we consider that bilateral partial adrenalectomy represents an appropriate therapeutic intervention for bilateral APAs, offering a high probability of achieving clinical and biochemical cures while simultaneously avoiding adrenocortical insufficiency.

With this case report, the possibility of bilateral APA should be considered when encountering PA patients with hypertension combined with spontaneous hypokalemia, high aldosterone levels, and bilateral solitary adrenal nodules in the clinic. ^68^Ga-Pentixafor nuclear imaging, which targets CXCR4, is a noninvasive and reliable method for PA subtyping and may be the optimal method for the definitive diagnosis of bilateral APA. In addition, bilateral partial adrenalectomy may be an effective and safe surgical procedure for the treatment of bilateral APAs. More in-depth studies on the diagnosis and treatment of patients with bilateral APAs are worthwhile in the future.

## Data Availability

The original contributions presented in the study are included in the article/supplementary material. Further inquiries can be directed to the corresponding author.
